# Social Characteristics, Health and Mortality among Male Centenarians using Veterans Affairs (VA) Healthcare

**DOI:** 10.1093/geroni/igaa057.3388

**Published:** 2020-12-16

**Authors:** Lien Quach David Gagnon, Elizabeth Dugan, Kelly Cho, Michael Gaziano, Avron Spiro, Rachel Ward, Jane Driver

**Affiliations:** 1 Boston University School of Public Health, Boston, Massachusetts, United States; 2 University of Massachusetts Boston, Boston, Massachusetts, United States; 3 VA Boston Healthcare System, Boston, Massachusetts, United States

## Abstract

Studying health and mortality among centenarian Veterans is critical to understanding the limit of the male human life span and Veterans’ extraordinary model of successful aging. The majority of VA users are male, but little is known about social characteristics and health among male centenarians in general. We investigated the annual mortality rate of male centenarian Veterans seeking care from the VA and identified social characteristics and health conditions that influenced the risk of mortality. This longitudinal study used VA Electronic Health Record (EHR) data from 1997 – 2012 (n=1858). Dates of death were obtained from the EHR, aggregated by the Corporate Data Warehouse from multiple sources. Independent variables included age, race, marital status, and periods of military service. Health conditions consisted of cancer, congestive heart failure (CHF), diabetes, chronic renal disease, chronic pulmonary disease, peripheral vascular disease, dementia, myocardial infarction, liver diseases. The mean age was 100.4 (range: 100-115), 76% were white and 49% married. The average annual mortality rate was 32 per 100 person-years. The annual mortality rate was stable and not affected by race, but did differ by marital status. Divorced or separated centenarians had a 21% higher rate of death than married centenarians [Hazard Ratio (HR):1.21, 95% CI 1.07 - 1.36]. A diagnosis of dementia increased the mortality risk by 37% (HR: 1.37, 95% CI: 1.04 - 1.81) and CHF by 37% (HR: 1.37; 95% CI: 1.13 - 1.66). Providers should consider prevalent health conditions, as well as marital status, in managing care of centenarian veterans.

